# Behavioral and histological assessment of a novel treatment of neuroHIV in humanized mice

**DOI:** 10.21203/rs.3.rs-3678629/v1

**Published:** 2023-12-13

**Authors:** Andrew J. Levine, Chirag Thadani, Virawudh Soontornniyomkij, Manuel F. Lopez-Aranda, Yoelvis Garcia Mesa, Scott Kitchen, Valerie Rezek, Alcino Silva, Dennis L. Kolson

**Affiliations:** University of California, Los Angeles; University of California, Los Angeles; University of California, San Diego; University of California, Los Angeles; University of Pennsylvania; UCLA Humanized Mouse Core Laboratory, University of California; UCLA Humanized Mouse Core Laboratory, University of California; University of California, Los Angeles; University of Pennsylvania

**Keywords:** HIV-associated neurocognitive disorder, CDDO-Me, Bardoxolone, humanized mouse, HIV, antiretroviral therapy

## Abstract

Neurocognitive deficits are prevalent among people living with HIV, likely due to chronic inflammation and oxidative stress in the brain. To date, no pharmaceutical treatments beyond antiretroviral therapy (ARV) has been shown to reduce risk for, or severity of, HIV-associated neurocognitive disorder. Here we investigate a novel compound, CDDO-Me, with documented neuroprotective effects via activation of the nrf2 and inhibition of the NFkB pathways.

**Methods:**

We conducted three studies to assess the efficacy of CDDO-Me alone or in combination with antiretroviral therapy in humanized mice infected with HIV; behavioral, histopathological, and immunohistochemical.

**Results:**

CDDO-Me in combination with ARV rescued social interaction deficits; however, only ARV was associated with preserved functioning in other behaviors, and CDDO-Me may have attenuated those benefits. A modest neuroprotective effect was found for CDDO-Me when administered with ARV, via preservation of PSD-95 expression; however, ARV alone had a more consistent protective effect. No significant changes in antioxidant enzyme expression levels were observed in CDDO-Me-treated animals. Only ARV use seemed to affect some antioxidant levels, indicating that it is ARV rather than CDDO-Me that is the major factor providing neuroprotection in this animal model. Finally, immunohistochemical analysis found that several cellular markers in various brain regions varied due to ARV rather than CDDO-Me.

**Conclusion:**

Limited benefit of CDDO-Me on behavior and neuroprotection were observed. Instead, ARV was shown to be the more beneficial treatment. These experiments support the future use of this chimeric mouse for behavioral experiments in neuroHIV research

## Introduction

Human immunodeficiency virus type1 (HIV) infection can result in significant neurobehavioral impairments. Among people with HIV (PWH), the spectrum of cognitive and functional deficits is termed HIV-associated neurocognitive disorders (HAND), which range from mild neurocognitive deficits, with limited impact on day-to-day functioning, to a rare and debilitating dementia(Antinori et al., 2007). Estimates vary widely, from 40%–60% in case-control studies (Heaton et al., 2011; McArthur et al., 2005) to less than 8% in cohort studies (Sacktor et al., 2016; Vance et al., 2016; Wang et al., 2021; Wang et al., 2019) within the United States, where antiretroviral (ARV) therapy is widely available and serves as the only effective prophylactic measure against the most severe forms of HAND(Kolson, 2022b). Considering that there are over one million PWH in the United States and almost 40 million worldwide, including large regions where access to ARV is problematic, HAND represents a significant public health concern both locally and globally.

While a complete understanding of HAND pathogenesis is lacking, the role of monocytes is well-documented. Both infected and uninfected monocytes cross the blood-brain barrier, driven by both increased chemokine release in the central nervous system (CNS) and an enhanced peripheral immune response (Ancuta et al., 2004; Kraft-Terry et al., 2009; Peluso et al., 1985). This increased trafficking of monocytes into the CNS further increases the expression of chemokines, promoting the migration of additional monocytes in a feed-forward manner (Persidsky et al., 1999; Persidsky et al., 2000). Once inside the CNS, monocytes differentiate into perivascular macrophages (Cosenza et al., 2002), where their role in the HAND pathogenesis includes the release of pro-inflammatory cytokines, chemokines, reactive oxygen species, interferons, and viral proteins, each of which can injure neighboring cells (Adle-Biassette et al., 1999; Glass et al., 1995; Kaul & Lipton, 2006; Kedzierska & Crowe, 2002; Kraft-Terry et al., 2009; Lindl et al., 2007). Clinico-pathological analysis has found that the density of perivascular macrophages is strongly associated with neurocognitive status, further implicating this mechanism a key driving force behind HAND (Glass et al., 1995).

Initially, a subset of monocytes was implicated in the severe form of HAND (i.e., HIV-associated dementia) (Ellery et al., 2007; Pulliam et al., 1997); however, more recent studies indicated that milder forms of HAND were the result of chronic neuroinflammation and low-grade viral replication driven in part by monocyte influx into the CNS (Langford et al., 2003; Yadav & Collman, 2009). In addition to inflammation, oxidative stress in the brain is an early and persistent consequence of HIV infection (Louboutin et al., 2010; Nath, 2002; Ronaldson & Bendayan, 2008), leading to neuronal dysfunction and death. Oxidative stress as a contributor to the pathogenesis of HAND through its effects on the brain is well established (Aksenov et al., 2001; Aksenov et al., 2003; Aksenova et al., 2006; Buckley et al., 2021; Sacktor et al., 2004; Toborek et al., 2003). In addition, HIV-induced oxidative stress may promote HAND pathogenesis via mechanisms outside of the CNS via activation of peripheral blood monocytes (Levine et al., 2013a; Williams et al., 2014). *In vitro* exposure of monocytes to HIV induces a proteomic response characterized by cellular activation and oxidative stress (Kadiu et al., 2009). Further, human studies have provided evidence for direct links between oxidative stress in peripheral blood monocytes and HAND. For example, anti-oxidant activity is diminished in monocytes and the cerebrospinal fluid of women living with HIV with HAND (Velazquez et al., 2009). Findings from a large gene expression study of participants in the Multicenter AIDS Cohort Study indicate that oxidative stress within peripheral blood monocytes is strongly correlated with neurocognitive dysfunction in PWH (Levine et al., 2013b). In sum, oxidative stress acting on monocytes/macrophages both inside and outside of the CNS contributes to HAND pathogenesis; therefore, treatments that mitigate this response in both body compartments are likely to be more effective.

Under conditions of oxidative stress, a robust endogenous anti-oxidant stress response is induced throughout the body, including within the brain (Morris et al., 2019). This response is largely mediated through the transcriptional activation of a cis-regulatory element known as the antioxidant response element (ARE), which is located in the promoter region of numerous genes that modulate cytoprotective responses against oxidative injury. Regulation of this host response depends upon the nuclear transcription factor erythroid 2p45-related factor 2 (nrf2), which in turn is under tight regulation by the nrf2 binding protein Kelch-like erythroid CNC homologue (ECH)-associated protein 1 (KEAP1). KEAP1 normally binds to and sequesters nrf2 in the cytoplasm, where it cannot act as a transcriptional activator (McMahon et al., 2003). Under oxidative stress, KEAP1 detaches from nrf2, translocates into the cell nucleus, and then binds to and transcriptionally activates the ARE in the promoter region of numerous antioxidant response genes (Ma, 2013). This ARE-modulated pathway is recognized as a target for neuroprotection (Barone et al., 2011; de Vries et al., 2008; Joshi & Johnson, 2012; Scapagnini et al., 2011; Tanji et al., 2013; van Muiswinkel & Kuiperij, 2005; Zhao et al., 2011), chemoprevention and chemoprotection (Lee & Surh, 2005; Surh et al., 2008; Surh et al., 2005), and anti-aging (Volonte et al., 2013). It has also been indirectly implicated as a target for HAND (Gill & Kolson, 2013; Kolson, 2022a) (Ambegaokar & Kolson, 2014; Garza et al., 2020; Gill et al., 2018), and among those molecules that inhibit the release of nrf2 is GSK3-β. GSK3-β inhibitors have shown modest promise for improving neurocognitive functioning in PWH (Ances et al., 2008; Letendre et al., 2006). Modification of KEAP1 functioning also influences transcriptional activity of NF-κβ (Itoh et al., 1999; Lv et al., 2013), the most potent inducer of HIV-1 replication and an inducer of inflammatory factors. Targeting NF-κβ and nrf2 via this pathway can both suppress pathological over-activation of NF-κβ signaling and activate cytoprotective genes (Wakabayashi et al., 2010; Z. Wang et al., 2014) (Cross et al., 2011). The relevance of the KEAP1/nrf2 pathway to HAND has been directly demonstrated; HIV-1 gp-120 upregulates nrf2 expression in human astrocytes, and this in turn stimulates gene and protein expression of the antioxidants heme oxygenase-1 (HO-1) and NAD(P)H quinone oxidoreductase-1 (NQO1). Further, expression of proinflammatory factors TNF-α, NF-κβ, and matrix metalloproteinase-9 is elevated in astrocytes in which nrf2 expression is suppressed (Reddy et al., 2012; Reddy et al., 2011), and upregulation of nrf2 protein expression in astrocytes reduces oxidative damage (Reddy et al., 2010). Nrf2 was also found to be suppressed in HIV transgenic rats as demonstrated by decreased nrf2 and HO-1 expression (Davinelli et al., 2014). It is also notable that HO-1 expression is induced via the KEAP1/nrf2 pathway (McDonagh, 1990; Ryter & Choi, 2009). HO-1 is highly expressed in CNS cells (primarily astrocytes, macrophages, microglia, and endothelia) particularly during brain injury in several neurodegenerative disease states (Browne et al., 1999; Castellani et al., 1995; Castellani et al., 1996; Fagone et al., 2013; Mateo et al., 2010; Mehindate et al., 2001; Schipper et al., 1995; Schipper et al., 1998; Smith et al., 1994; Takeda et al., 2000). HO-1 is significantly reduced in prefrontal cortex of PWH with HAND (Gill et al., 2014), and this HO-1 deficiency correlates with brain HIV-1 RNA load, macrophage activation, and type-I interferon response. Further, an *in vitro* HIV neurotoxicity model demonstrates that HIV infection of monocyte-derived macrophages markedly reduces HO-1 expression and that this deficiency is linked to release of glutamate, a HAND-associated excitotoxin (Drummond et al., 1987; Huang et al., 2011; Jiang et al., 2001). This HO-1 deficiency and associated excitotoxin production are a generally conserved feature of infection with macrophage-tropic HIV-1 strains and correlate closely with the extent of virus replication (Gill et al., 2015), suggesting a potential therapeutic benefit of restoring HO-1 expression in HIV-infected brain macrophages. Induction of HO-1 expression in HIV-infected macrophages suppresses the release of excitotoxic levels of glutamate and thereby protects neurons against HIV-induced excitotoxic injury (Gill et al., 2014). Considering these findings from a variety of independent groups of investigators and research models, therapeutics that activate the KEAP1/nrf2 pathway as a preventative measure for HAND are worth examination.

One such nrf2 pathway activator is Bardoxolone methyl ester (CDDO-Me), an orally-available semi-synthetic triterpenoid that acts as an activator of the nrf2 pathway and an inhibitor of the NF-κB pathway. Several preclinical and Phase I and Phase II clinical studies have delineated the mechanistic, safety, and tolerability aspects of this compound (Hong et al., 2012; Y. Y. Wang et al., 2014; Warnock et al., 2012). Phase III clinical trials of CDDO-Me continue for other conditions such as pulmonary arterial hypertension and chronic kidney disease. Based on its mechanism of action, the studies described above suggest that CDDO-Me holds promise for preventing or treating HAND. Our present study described several experiments engineered to test the hypothesis that CDDO-Me could prevent or treat HAND in PWH. We first assessed the effects of oral CDDO-Me administration on behavioral functioning of humanized mice engineered to express human monocytes and T-cells. We then examined histopathology and expression of neuroprotective factors (e.g., HO-1) in the mouse brains. Our hypothesis was that CDDO-Me administration following HIV-infection would prevent HIV-induced behavioral deficits and neuropathology via increases in neuroprotective factors.

## METHODS

The Chancellor’s Animal Research Committee at the University of California, Los Angeles, approved the research protocols described here.

### HIV Infection of Humanized Mice

We used NOD.Cg-*Prkdc*^*scid*^ *Il2rg*^*tm1Wjl*^/SzJ (NSG) immunodeficient mice. Two sets of mice were produced using fetal liver-derived CD34^+^ cells (2.5 to 5 ×10^5^) from 2 donors. Fetal liver CD34 + cells were transplanted into irradiated newborn NSGs mice 3–5 days post birth by intrahepatic injection. After reconstitution, mice were intravenously infected with R5-tropic HIV-1 (NFNSXSL9), generated by transfection of 293T cells with plasmid containing full-length HIV-1 (NFNSXSL9) genome. The humanized mice were infected with NFNSXSL9 (400 ng of p24 per mouse) through retro-orbital injection while under inhalant general anesthesia. Infected mice with demonstrable viral infection were treated for 12 weeks with ARV drugs. Plasma HIV-1 RNA loads were monitored by quantitative real-time RT-PCR every two weeks. Chronic HIV infection was generally established 4–6 weeks following viral exposure. The ARV regimen consisted of tenofovir disproxil-fumarate (TDF, 80 mg/kg), emtricitabine (FTC, 120 mg/kg), and Elvitegravir (ELV, 160 mg/kg) given by food. These medicines were generously supplied by Gilead Sciences. TDF, FTC, and ELV were dissolved in DMSO and mixed with sweetened moist gel meal (DietGel Boost, ClearH2O; Medidrop Sucralose). CDDO-Me was administered via drinking water (10 mg/kg) (Saha et al., 2010), as described below.

### Behavioral Testing of HIV-Infected Humanized Mice

The mice were initially grouped and treated as follows:

1. Pre-suppression ARV + CDDO-Me: Daily oral administration of CDDO-Me (10 mg/kg) via drinking water beginning immediately after viral setpoint was reached along with suppressive ARV therapy until viral suppression was reached, typically after 4 weeks.
2. Post-suppression ARV + CDDO-Me: The same approach as above, but with CDDO-Me administration beginning after viral suppression had occurred, typically 4 weeks after ARV initiation. Once viral loads were determined to be below the detection levels, treatment with a daily oral administration of CDDO-Me (10 mg/kg) in drinking water was initiated and continued along with the suppressive ARV therapy for 10 weeks.
3. ARV + Placebo condition: Same as above but without CDDO-Me.
4. No ARV + Placebo: DietGel Booast without ARV therapy or CDDO-Me.

Behavioral functioning was assessed via four tests: Social Interaction Test (SIT), Social Recognition Memory Test (SMT), Novel Object Recognition (NOR), and Object Place Recognition (OPR). Details of these tests can be found in Supplemental Materials. We have previously shown these tests to share characteristics of other hippocampus-dependent spatial and object memory (Kogan et al., 2000), and similar tests have previously been used in HIV-related mouse studies (Kalkonde et al., 2011; Kesby et al., 2015; Soontornniyomkij et al., 2016). For each experimental group, we compared exploration time in familiar vs. novel conditions 24-hours after training. Specifically, exploration of a familiar vs. novel mouse (in SMT), object (in NOR), and location (in OPR). Mice underwent training and testing at 7 months of age, 4 months after HIV infection and treatment. Prior to testing, mice were handled daily for eight minutes over six days, followed by two days of habituation to a three-chamber open field (63.6 × 43 × 24.5 cm) for 12 minutes each day. All tests were run consecutively over approximately one week, beginning with SIT and SMT, followed by NOR and OPR. Stimulus location was randomly alternated between left and right. Background noise, humidity, and 70% ethanol scent were kept consistent throughout the habituation and test days. Testing apparatus was centered on a lab bench to minimize gradients in light, temperature, and other environmental conditions that could produce a side preference. All equipment was cleaned between conditions with 70% isopropyl alcohol to remove residue scents. Exploration times were calculated by the same human observer (C.T.) across all animals, and validated with video recordings. The observer was blind to the mouse group membership. Additional information concerning the behavioral tests can be accessed in Supplemental Materials.

### Histopathological and Western Blot Analyses of Mouse Brains

This experiment assessed the efficacy of CDDO-Me for increasing nrf2-induced cytoprotective factors and for mitigating HIV-induced injury in humanized mouse brains. Mice were sacrificed at approximately 7–8 months of age, immediately after completing behavioral testing. A final blood draw was taken, spleens removed, blood vessels tied off, and the animals underwent a saline perfusion protocol prior to brain removal. Brain hemispheres were then separated. One hemisphere was flash-frozen in isopentane, stored at −80°C until being shipped on dry ice in one complete batch to the lab of DLK. After thawing, each specimen was dissected for five brain regions (frontal cortex, parietal cortex, occipital cortex, striatum, and hippocampus) (Spijker, 2011). Brain tissue lysates were prepared by homogenization (~ 100 mg of tissue) by silica bead beating and sonication in 5 volumes buffer (10 mM Tris-HCl pH 7.8, 0.5 mM Dithiothreitol, 5 mM MgCl_2_, 0.03% Triton X-100) containing a phosphatase inhibitor cocktail set II (EMD Millipore) and a protease inhibitor cocktail (Sigma-Aldrich). Protein was quantified using the DC^™^ (detergent compatible) protein assay (Bio-Rad). Equivalent amounts of proteins were added to Laemmli sample buffer (50 mM Tris-HCl pH 6.8, 2% SDS, 10% glycerol, 12.5 mM EDTA, 0.002% bromophenol blue) with 2.5% 2-Mercaptoethanol and denatured at 95°C for 10 minutes. Proteins were resolved on an SDS-PAGE gel and transferred over night to poly(vinylidene fluoride) (PVDF) membranes (4°C). Membranes were blocked with Odyssey Blocking Buffer (PBS) (LI-COR Biosciences) and incubated with primary antibody overnight (4°C). TRDye-conjugated secondary antibodies (LI-COR Biosciences) were used to detect the primary antibody. Quantification of protein bands was determined using Image Studio Lite software (LI-COR Biosciences). One sample prepared from mixing equal volumes of all samples (Mix) was used as running and transfer control in all membranes. Each blot was normalized to that sample in each membrane making possible to compare all brain regions and animals (Garcia-Mesa et al., 2020a). The antibodies used in this study are listed in Supplemental Materials. To assess neuronal injury, antioxidant protein expression, and potential protection by ARV, with and without CDDO-Me, we quantified expression of postsynaptic density-95 (PSD-95 (Garcia-Mesa et al., 2020a)), heme oxygenase-1 and − 2 isoforms (HO-1, HO-2, respectively), glutathione peroxidase 1 (GPX1), glutathione peroxidase 4 (GPX4), and peroxiredoxin 1 (PRDX1) proteins by Western blotting in the five brain regions listed above. We have used this approach (individual brain regions and grouped brain regions) to quantify synaptic injury and recovery associated with simian immunodeficiency virus (SIV) infection in individual and grouped brain in SIV-infected rhesus macaques (Garcia-Mesa et al., 2020a).

For histopathological analysis, the remaining brain hemisphere was fixed in 4% paraformaldehyde/PBS (4°C, 3 days) at the time of animal sacrifice. The brain samples were sent to the lab of VS for evaluation of synaptodendritic degeneration with antibodies against synaptophysin (SYP) and microtubule associated protein 2 (MAP2); gliosis with antibodies against glial fibrillary acidic protein (GFAP) and ionized calcium-binding adaptor molecule 1 (IBA1) and HIV-1 p24 burden in the hippocampus (dorsal hippocampal formation), striatum, and frontal cortex separately on parasagittal brain sections (Soontornniyomkij et al., 2012). Hematoxylin and eosin histopathology and chromogenic (3,3’-diaminobenzidine, DAB) immunohistochemistry were conducted on adjacent 5-μm-thick paraffin-embedded tissue sections (two technical replicates on single microscope slides). By means of two-dimensional computer-assisted image analysis, immunoreactivity for SYP, MAP2, GFAP, and IBA1 was quantified on DAB tissue slides. In brief, the brain sections were digitally scanned with a microscope slide scanner (Aperio ScanScope GL, Leica Biosystems, Buffalo Grove, IL, USA) equipped with a (doubled) 20x objective lens. Using Aperio ImageScope software, the hippocampus, striatum, and frontal cortex separately were digitally drawn on each of brain images. For each marker, color segmentation was set to select the specific signal and then consistently applied to all the brain samples based on the Aperio Positive Pixel count algorithm. The quantitative analysis results were used to calculate the immunoreactivity density, i.e., ([0.75 × Number of Weak Positive] + [Number of Moderate Positive] + [1.25 × Number of Strong Positive]) / Area (μm^2^). For each brain sample, the average immunoreactivity density of two technical replicates was used for data analysis. The investigators conducting histopathological assessments of the hemispheres were blind to the experimental conditions. See Supplemental Materials for detail.

### Statistical Analysis

#### Behavioral testing:

Two-way ANOVA and t-test were performed to compare the exploration times among 3 groups between the two objects on the test day. Due to small group numbers resulting from mouse attrition, the 2 CDDO-Me treatment groups were combined, resulting in three groups: No ARV + Placebo (5 animals: 2 males, 3 females), ARV + Placebo (9 animals: 3 males, 6 females), and ARV + CDDO-Me (19 animals: 9 males, 10 females). Data were expressed as % of total exploration time. *For Experiment 2*, we maintained the original 4 groups. We performed two-way ANOVA, with sex and treatment group as covariates using the lm routine of the R statistical package (version 4.0.3). We tested 24 outcomes and used an FDR of 0.05 as our significance cutoff to adjust for multiple comparisons.

#### Western blotting:

A processed brain tissue ‘mix’, sample made by mixing equal volumes of all samples was used as a running and transfer control in all membranes. Each blot was normalized to that sample in each membrane, allowing for comparisons between all brain regions and animals. Tubulin was used as a loading control in all membranes. Using GraphPad Prism software, Western blot band signal intensities were compared by two-way ANOVA with repeated measures and Tukey’s multiple comparisons. Values were expressed as means with standard errors of the means. For these analyses, we preserved the original four groups.

#### Histopathological analysis:

Two-way ANOVA were performed to compare the absolute levels of IBA-1, GFAP, MAP2, and SYP in each of three regions between the three groups, as described above.

## RESULTS

### Behavioral testing reveals rescue of social interaction deficits in animals receiving ARV and CDDO-Me

**FIGURE 1:**
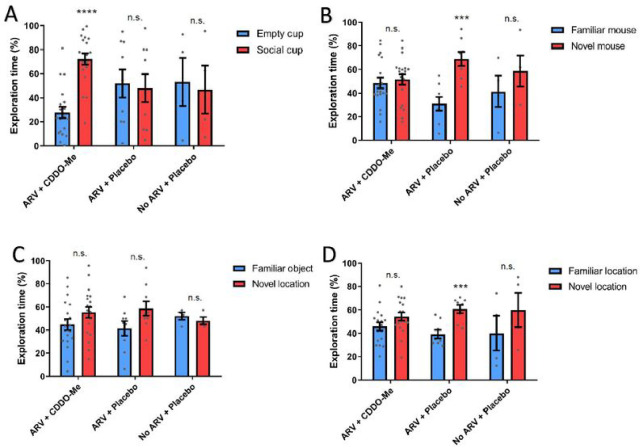
Panel A - Social Interaction: The graph shows the % of time that mice spent actively exploring the empty cup and the social cup. ARV+CDDO-Me mice spent significantly more time exploring the social cup than the empty cup (n=19; P<0.0001, t=6.48). The ARV+Placebo (n=9; P=0.81, t=0.23) and No ARV+Placebo (n=4; P=0.83, t=0.22) mice do evince significantly different percentage of time exploring the social cup and empty cups. **Panel B - Social Recognition Memory:** The graph shows the % of time that mice spent actively exploring the cup with the familiar mouse and the cup with the novel mouse. The ARV+CDDO-Me (n=19; P=0.63, t=0.47) and No ARV+Placebo (n=4; P=0.39, t=0.92) mice did not evince significant differences in time spent exploring the novel and familiar mice. The ARV+Placebo mice spent significantly more time exploring the novel mouse than the familiar mice (n=8; P<0.001, t=4.56). **Panel C - Novel Object Recognition:** The graph shows the % of time that mice spent actively exploring the familiar object and the novel object. None of the groups spent significantly different time exploring the novel and familiar objects: ARV+CDDO-Me (n=19; P=0.13, t=1.54); ARV+Placebo (n=9; P=0.071, t=1.92); No ARV+Placebo (n=4; P=0.39, t=0.9). **Panel D - Object Place Recognition:** The graph shows the % of time that mice spent actively exploring the object in the familiar location and the object in the novel location. The ARV+CDDO-Me (n=18; P=0.0.94, t=1.71) and No ARV+Placebo (n=4; P=0.38, t=0.94) mice did not spend more time exploring the object in the novel location compared to the object in the familiar location. The ARV+Placebo mice did spend significantly more time exploring the object in the novel location than the object in the familiar location (n=8; P<0.0001, t=4.16). For all graphs, data represent means ± SEM as well as individual data

Social Interaction-SIT: Animals within the ARV + CDDO-Me group spent significantly more time exploring the social cup compared to the empty cup (n = 19; P < 0.0001, t = 6.48) (Fig. 1A, data expressed as % of total exploration time). No differences in time exploring the social and empty cup were observed for either the ARV + Placebo group (n = 9; P = 0.81, t = 0.23) or the No ARV + Placebo group (n = 4; P = 0.83, t = 0.22). These results suggest that the combination of ARV + CDDO-Me is able to rescue social interaction deficits in the HIV-infected humanized mouse model.

#### Social Recognition Memory-SMT:

In contrast with the Social Interaction-SIT test, the Social Recognition Memory-SMT test showed no significant different effect on animals within the ARV + CDDO-Me group (n = 19; P = 0.63, t = 0.47) or No ARV + Placebo group (n = 4; P = 0.39, t = 0.92) (Fig. 1B). However, the ARV + Placebo group did spend significantly more time exploring the novel mouse than the familiar mouse (n = 8; P < 0.001, t = 4.56). These results suggest that ARV treatment is able to rescue the social memory deficits shown in our mouse model. Nonetheless, the administration of CDDO-Me may have attenuated the positive effect of ARV in social memory.

#### Novel Object Recognition-NOR:

No significant effect on Novel Object Recognition (NOR) was observed, as none of the grouped animals explored the novel object significantly more than the familiar object: ARV + CDDO-Me (n = 19; P = 0.13, t = 1.54); ARV + Placebo (n = 9; P = 0.071, t = 1.92); No ARV + Placebo (n = 4; P = 0.39, t = 0.9) (Fig. 1C). These results indicate that neither the administration of ARV alone nor ARV + CDDO-Me is able to rescue the novel object recognition deficits in our mouse model.

#### Object Place Recognition-OPR:

A positive effect of ARV was observed in the Object Place Recognition-OPR test (Fig. 1D). The ARV + Placebo (n = 8; P < 0.001, t = 4.16) group spent significantly more time exploring the object in the novel location than the object in the familiar location. In contrast, neither the ARV + CDDO-Me group (n = 18; P = 0.94, t = 1.71) nor the No ARV + Placebo group (n = 4; P = 0.38, t = 0.94) spent more time exploring the object in the novel location compared to the object in the familiar location. These results support the idea that ARV treatment is able to rescue the spatial memory deficits shown in our mouse model. However, similar to results observe in Social Recognition Memory-SMT test (Fig. 1B), the administration of CDDO-Me may have attenuated the beneficial effect of ARV in this test.

### Limited preservation of PSD-95 by CDDO-Me treatment in animals receiving ARV as evidence for neuroprotection.

We used western blotting to quantify expression of PSD-95 as a marker of postsynaptic neuronal structural integrity and multiple antioxidant enzymes as markers of the host anti-oxidant response. This combined analysis provides an assessment of potential neuroprotection afforded by ARV and CDDO-Me treatment. Antioxidant enzymes examined include HO-1, HO-2, GPX1 and GPX4 (Huang et al., 2018), and PRDX1 (Lee, 2020). Among these enzymes each is driven at least in part by nrf2, except for HO-2, which is constitutively expressed and which expresses the same enzymatic function as HO-1 (Ma, 2013). Overall, enhanced expression of these antioxidant enzymes is associates with effective cytoprotection.

**Figure 2:**
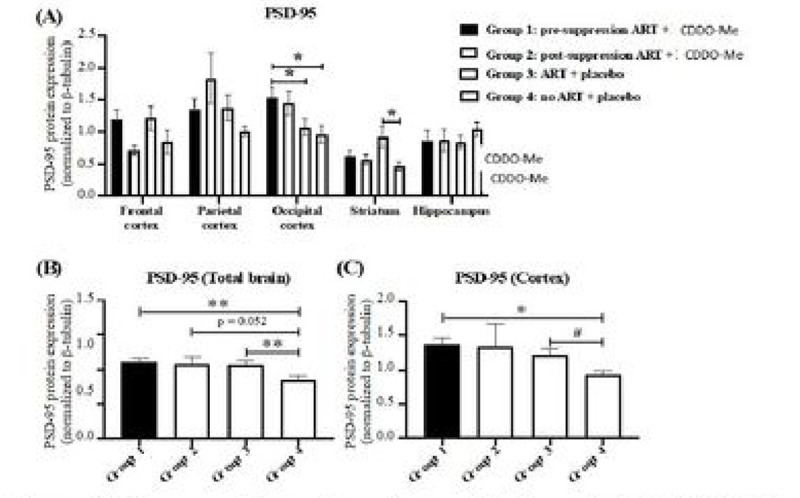
Preservation of postsynaptic density-95 (PSD-95) neuronal protein in the brains of ARV-treated, HIV-1-infected mice. **(A)** PSD-95 expression is significantly higher in occipital cortex in mice receiving concurrent CDDO-Me + ARV when compared to ARV + placebo or to no ARV + placebo. **(B)** Total brain and **(C)** cortical PSD-95 expression are significantly higher in mice receiving concurrent CDDO-Me + ARV or ARV + placebo when compared to no ARV + placebo. Values are the mean ± SEM.

Initiation of CDDO-Me treatment concurrently with ARV, but not after ARV-induced HIV suppression, was associated with preserved PSD-95 expression in total brain and grouped cortex (frontal, parietal, occipital) regions (Fig. 2). This neuroprotective effect of ARV + CDDO-Me was most pronounced in the occipital cortex and was not observed in the striatum or hippocampus (Fig. 2A). When regional brain protein expression values for all regions were averaged (‘total brain’), or as ‘cortex’ (frontal, parietal, and occipital), a pattern of preserved PSD-95 expression under conditions of concurrent initiation of ARV and CDDO-Me (group 1) or ARV and placebo (group 3) was observed (Fig. 2B, C).

### No effect of CDDO-Me treatment on expression of antioxidant enzymes.

**Figure 3.**
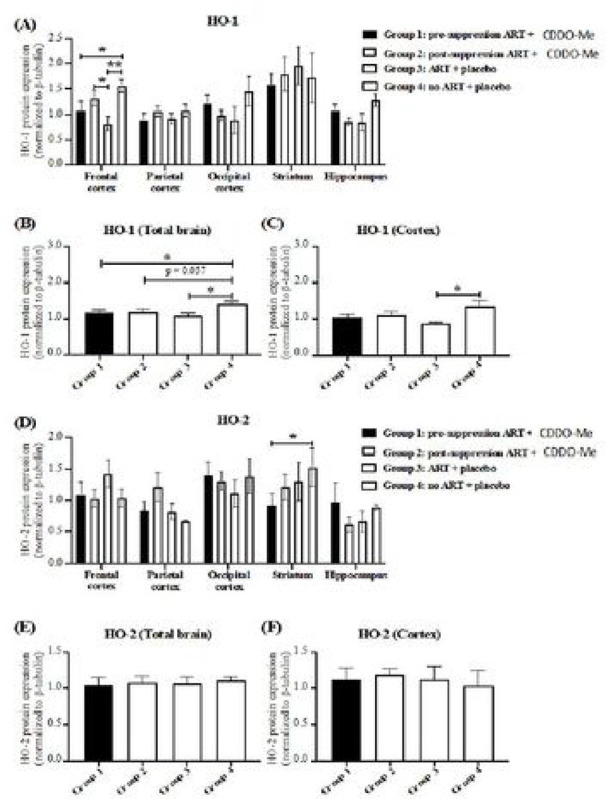
Expression of heme oxygenase isoforms 1,2 (HO-1, HO-2) in the brains of ARV-treated, HIV-1-infected mice. **(A)** HO-1 expression is higher in frontal cortex in untreated animals compared with those receiving ARV + placebo, or concurrent ARV + CDDO-Me. **(B)** Similarly. HO-1 was higher in untreated animals’ total brain and (C) grouped cortex (frontal, parietal, occipital) regions. HO-2 was elevated only in the striatum in untreated animals **(D),** and not elsewhere **(E, F).**

We next examined expression of heme oxygenase (two isoforms, HO-1 and HO-2). The HO-1 isoform is robustly inducible in response to oxidative stress, and is modestly inducible by nrf2 activating agents (Abraham & Kappas, 2008). In contrast, the HO-2 isoform is considered to be constitutively expressed, and not readily inducible. Figure 3 shows expression of each isoform in brain regions examined in Fig. 2. Expression of HO-1 was significantly higher in animals not receiving ARV when compared to those receiving ARV with or without CDDO-Me. This significant difference was observed in frontal cortex when untreated animals were compared with those receiving ARV + placebo, or concurrent ARV + CDDO-Me (Fig. 3A). Significantly higher HO-1 expression was also observed in untreated animals in total brain and grouped cortex (frontal, parietal, occipital) regions (Fig. 3B, C). In similar analyses of the constitutive isoform HO-2 was significantly elevated only in the striatum in untreated animals (Fig. 3D), and not elsewhere (Fig. 3E, F). These data suggest that unsuppressed HIV-1 replication, in the absence of ARV, may enhance endogenous expression of HO-1.

**Figure 4.**
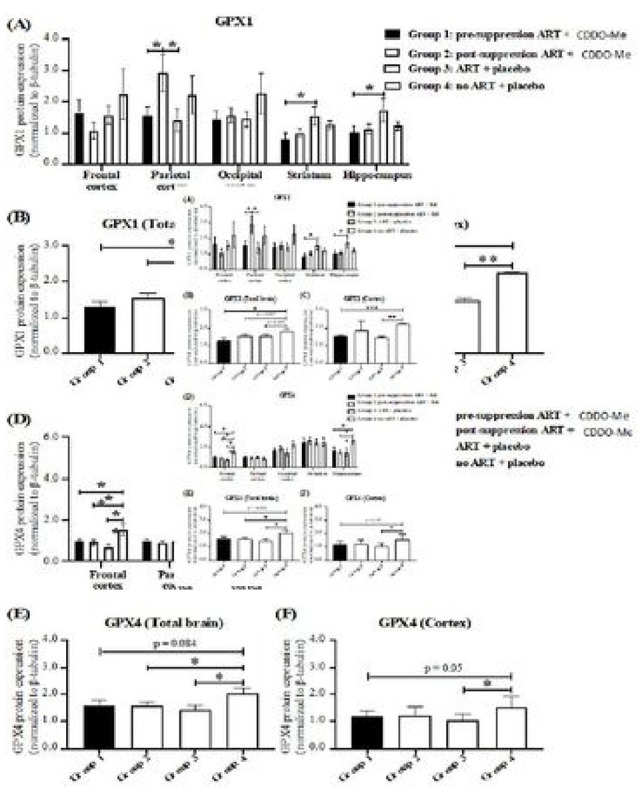
Expression of GPX1 and GPX4 in the brains of ARV-treated, HIV-1-infected mice. **(A)** GPX1 expression is higher in parietal cortex, striatum, and hippocampus in ARV-treated animals compared to untreated animals. **(B)** GPX1 was higher in untreated animals’ total brain and (**C)** grouped cortex (frontal, parietal, occipital) regions. GPX4 was elevated in frontal cortex and hippocampus (D). and in total brain (E) and grouped cortex (F) only in untreated animals.

Similarly, expression of GPX1 and GPX4 was not altered by CDDO-Me treatment (Fig. 4). GPX1 expression varied significantly in several brain regions (Fig. 4A). Similar to HO-1, GPX1 was also significantly higher in untreated animals in total brain and grouped cortex (frontal, parietal, occipital) regions compared to all treatment conditions (Fig. 4B, C). In contrast, GPX4 expression was elevated only in untreated animals when examined regionally (Fig. 4D), in total brain (Fig. 4E) or in grouped cortex (frontal, parietal, occipital) regions (Fig. 4F).

**Figure 5.**
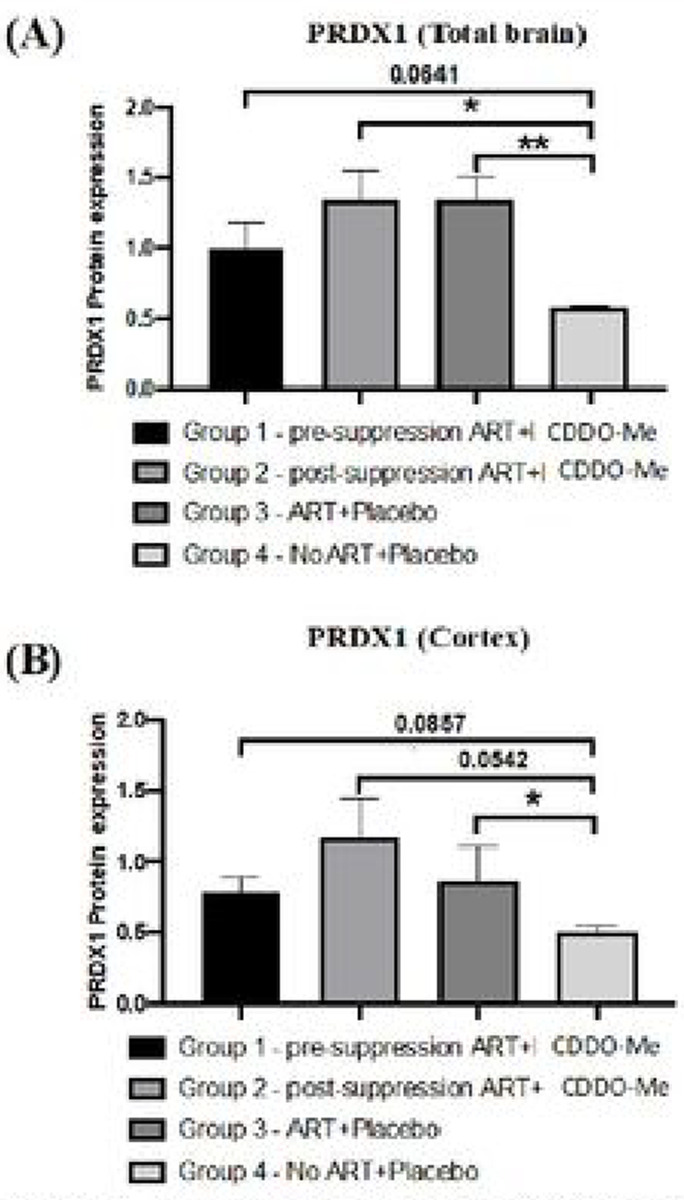
Expression of PRDX1 in the brains of ARV-treated, HIV-1-infected mice. (A). PRDX1 expression. PRDX1 expression was lower in total brain **(A)** and in grouped cortex (frontal, parietal, occipital regions **(B)** in untreated animals compared to ARV-treated animals. Two-way ANOVA with mixed-effects analysis Tukey’s multiple comparison test.

Finally, and in contrast, PRDX1 expression was significantly lower in animals not receiving ARV when compared to those receiving ARV with or without CDDO-Me (Fig. 5)

### Histopathological differences were due to ARV treatment, not CDDO-Me

P-values provided in Table 1 are the nominal per test p-values. With regard to treatment effects, four of the outcomes were significant at an FDR of 0.05. These were: hippocampus GFAP, striatum IBA1, hippocampus IBA1, and frontal cortex IBA1. For these 4 outcomes, we compared the individual treatment groups in post-hoc analyses. We determined that for all 4 outcomes, the difference was due to the effect of ARV and not to CDDO-Me. Animals treated with ARV had increased IBA1 values (~ 0.15) over animals without treatment. Animals treated with ARV and CDDO-Me had essentially the same IBA1 values as animals treated with ARV alone. We observed no sex effects on any of the outcomes (data not shown).

## DISCUSSION

HIV infection can result in significant neurobehavioral impairments that may be driven by neuroinflammation, oxidative stress and other HIV-induced pathogenic responses in the brain. We hypothesized that such deficits in the HIV-1-infected humanized mouse model are driven in large part by oxidative stress in the brain, and that this could be counteracted by inducing the KEAP/nrf2 transcription pathway of antioxidant responses. We further hypothesized that CDDO-Me, a potent inducer of nrf2, would serve as a prophylactic agent to prevent HIV-induced neuropathology and behavioral changes in these mice, as supported by several lines of research (Ances et al., 2008; Barone et al., 2011; Davinelli et al., 2014; de Vries et al., 2008; Gill et al., 2014; Gill et al., 2015; Itoh et al., 1999; Joshi & Johnson, 2012; Letendre et al., 2006; Levine et al., 2013b; Lv et al., 2013; McDonagh, 1990; Reddy et al., 2012; Reddy et al., 2011; Reddy et al., 2010; Ryter & Choi, 2009; Scapagnini et al., 2011; Tanji et al., 2013; van Muiswinkel & Kuiperij, 2005; Wakabayashi et al., 2010; Z. Wang et al., 2014; Zhao et al., 2011).

Across four behavioral tasks, we observed that the combination of ARV + CDDO-Me was able to rescue only the social interaction deficits demonstrated by our No ARV + Placebo control group (Fig. 1A). Importantly, this social Interaction deficit was not rescued by the ARV treatment alone (Fig. 1A). Conversely, CDDO-Me treatment did not rescue memory deficits, including the social recognition (Fig. 1B), novel object recognition (Fig. 1C), or object place recognition (Fig. 1D) deficits exhibited by the control group. Notably, ARV treatment alone (without CDDO-Me) was able to rescue the social recognition (Fig. 1B) and object place recognition (Fig. 1D) deficits. These results suggest that the ARV treatment has a positive impact in hippocampal dependent tasks such as social recognition and object place recognition, but not in cortical dependent tasks such as novel object recognition, in this humanized mouse model.

Notably, we observed some evidence for a neuroprotective effect (i.e., preservation of PSD-95 expression) of CDDO-Me when administered with ARV (Fig. 2A, occipital cortex), which was nonetheless not as consistent with the protective effect of ARV alone (Fig. 2B, C). Expression of PSD-95 is largely confined to the post-synaptic regions of neuronal synapses, and it is a sensitive marker for synapse function and integrity (Vallejo et al., 2017), and synaptic injury is a common neuropathological feature of HIV infection (Gelman & Nguyen, 2010). Whether this modest CDDO-Me effect is directly related to the rescue effect on the social interaction behavioral test is unknown. Furthermore, neither of the effects observed with CDDO-Me treatment can be attributed to induction of antioxidant gene responses, as no significant changes in antioxidant enzyme expression levels (HO-1, HO-2, GPX1, GPX4) were observed in CDDO-Me-treated animals, with the exception of reduced expression of PRDX1.

The significantly higher levels of HO-1, GPX1 and GPX4 in animals not receiving ARV with or without CDDO-Me may reflect the natural host endogenous antioxidant response to unsuppressed HIV infection in the brain (Gill et al., 2014; Gruenewald et al., 2020). Such responses (induction of antioxidant response enzymes, particularly HO-1) is observed in a variety of injurious insults. Our failure to observe even higher levels of enzyme expression in the presence of CDDO-Me may reflect our inability to detect any effects above that caused by HIV infection, lack of effective in vivo drug levels, or other unknown factors.

The significant decrease in PRDX1 expression in CDDO-Me treated animals without ARV administration (decreased striatum PRDX1 values ~ 1.1 lower) compared to mice treated with ARV alone indeed suggests a pharmacological effect within the brains of these animals. The remarkable discordance in expression between PRDX1 and the other enzymes in the setting of unsuppressed HIV infection suggests that responses to HIV infection itself (e.g., neuroinflammation) may be driving selective antioxidant gene expression. Previous studies have shown differential induction of PRDX1, HO-1, and other nrf2-driven antioxidant genes in response to pro-inflammatory signaling (Ingram et al., 2019). We have observed the same discordance in the relative expression levels of GPX1, HO-1, and PRDX1 in the brains of rhesus macaques with unsuppressed SIV infection (Garcia-Mesa et al., 2020b), which suggests a consistent association of these antioxidant enzyme expression patterns with both HIV and SIV infections in these model systems.

HO-1, GPX1, GPX4, and PRDX1 have both distinct and overlapping functions. For example, HO-1 and HO-2 break down free heme, a major intra- and extra-cellular pro-oxidant, into carbon monoxide, biliverdin, and ferrous iron, with cytoprotective effects in numerous model systems (Basuroy et al., 2006; Chang et al., 2003; Chen, 2014; Chen et al., 2005). GPX1 is a detoxifier of peroxides and a suppressor of reactive oxygen species production and oxidative damage (Huang et al., 2018). It is particularly important for mitochondrial H_2_O_2_ scavenging (Starkov et al., 2014). GPX4 is specifically important in suppressing lipid peroxidation in the cellular membrane, and it thereby is a key suppressor of cellular injury by ferroptosis (Conrad et al., 2018; Yang et al., 2014). PRDX1 also detoxifies peroxides, including H_2_O_2_, and peroxynitrite (Goemaere & Knoops, 2012), and it suppresses microglial activation (Kim et al., 2013; Neumann et al., 2009). Expression of each of these is regulated, at least in part, by the nrf2 transcription factor, in response to various cellular stressors. Despite this, we observed no differences in expression between CDDO-Me-treated and untreated animals. Only ARV use seemed to affect PRDX1 levels, indicating that it is ARV rather than CDDO-Me that is the major factor providing neuroprotection in this animal model. Because ARV suppression of HIV replication in the mice revealed a brain antioxidant expression pattern that likely reflects a consistent response to HIV infection in the brain, the findings support the use of this animal model for the further assessment of neuroprotection strategies based upon modulation of endogenous antioxidant enzyme responses.

Finally, immunohistochemical analysis found that several cellular markers in various brain regions differed between the groups: hippocampus GFAP and IBA1, striatum IBA1 and PRDX1, and frontal cortex IBA1. Similar to the behavioral and Western blot analyses, post-hoc analyses determined that the difference in the outcomes was largely due to the effect of ARV and not to CDDO-Me. Mice treated with ARV had increased IBA1 values (~ 0.15) over animals without treatment, and animals treated with ARV and CDDO-Me had essentially the same values as animals treated with ARV alone.

Several factors limit the interpretability and generalizability of our findings. Firstly, we lacked groups that might have been useful, including a CDDO-Me-only group and an HIV-uninfected group. The complexity of engineering the humanized mice limited the number of animals available, which was in fact lower than we had anticipated, thus resulting in the combining of the CDDO-Me treatment groups for the behavioral and histopathological analyses. Secondly, the behavioral experiments, while validated and useful, might not have captured underlying neuropathological processes in the mice. Future studies that employ a wider range of behavioral tests could be useful, but they also need to consider the relatively fragile health and short lifespans of humanized mice.

In summary, while we did observe significant benefit from CDDO-Me administration together with ARV in rescuing the social interaction deficits demonstrated in our humanized mouse model of neuroHIV, we did not observe a benefit from CDDO-Me administration in maintaining other behaviors. Further, CDDO-Me did not elicit neuroprotection against HIV-mediated neuropathology. ARV use, however, did seem to preserve cognitive functioning and cellular integrity. These experiments do support the future use of this chimeric mouse for behavioral experiments.

## Figures and Tables

**Table 1:** Results of Western Blot testing

Outcome	F	P-value	Significant at FDR of 0.05?	No Treatment versus ARV only (p-value)	CDDO-Me+ARV versus ARV only (p-value)
GPX1 Striatum	3.9832	0.02962	no		
GPX1 Hippocampus	2.2948	0.1188	no		
GPX1 Frontal Cortex	0.9262	0.4079	no		
HO-1 Striatum	0.3679	0.6954	no		
HO-1 Hippocampus	2.1276	2.1276	no		
HO-1 Frontal Cortex	4.9415	0.01452	no		
HO-2 Striatum	0.9744	0.3894	no		
HO-2 Hippocampus	0.1459	0.8649	no		
HO-2 Frontal Cortex	1.2625	1.2625	no		
PRDX1 Striatum	5.9664	0.006757	yes	−2.1026 (0.00236)	−1.1215 (0.02132)
PRDX1 Hippocampus	0.4346	0.6517	no		
PRDX1 Frontal Cortex	0.5469	0.5848	no		

GFAP = glial fibrillary acidic protein; GPX1 = glutathione peroxidase 1, HO-1 = heme oxygenase 1; HO-2 = heme oxygenase 2; IBA1 = ionized calcium-binding adaptor molecule 1; MAP2 = microtubule associated protein 2; PRDX1 = peroxiredoxin 1; SYP = synaptophysin.
